# Can Between-Session Homework Be Delivered Digitally? A Pilot Randomized Clinical Trial of CBT for Adjustment Disorders

**DOI:** 10.3390/ijerph16203842

**Published:** 2019-10-11

**Authors:** Soledad Quero, Iryna Rachyla, Mar Molés, Sonia Mor, Cintia Tur, Pim Cuijpers, Alba López-Montoyo, Cristina Botella

**Affiliations:** 1 Department of Basic, Clinical Psychology, and Psychobiology, Universitat Jaume I, 12006 Castellón, Spain; irinarachila@gmail.com (I.R.); molesm@uji.es (M.M.); smor@uji.es (S.M.); ctur@uji.es (C.T.); montoyo@uji.es (A.L.-M.); botella@uji.es (C.B.); 2 CIBER Fisiopatología de la Obesidad y Nutrición (CIBERON), Instituto Salud Carlos III, 28029 Madrid, Spain; 3 Department of Clinical, Neuro, and Developmental Psychology, Amsterdam Public Health Research Institute, Vrije Universiteit Amsterdam, 1081BT Amsterdam, The Netherlands; p.cuijpers@vu.nl

**Keywords:** CBT, between-session homework, Internet-delivered therapy, virtual reality, feasibility, efficacy, adjustment disorders

## Abstract

Adjustment disorder (AjD) is one of the most common disorders in clinical practice, and its symptoms are severe enough to cause great distress and functional impairment. The AjD CBT protocol specifically developed for this disorder has shown positive results when delivered face to face and through virtual reality. Despite existing evidence supporting the benefits of therapeutic homework as part of a psychological intervention, little is known about how to increase homework engagement in psychotherapy. This study examines the feasibility (doability, initial efficacy and acceptability) of a digital support system to deliver homework via the Internet in the treatment of AjD. Participants were randomly assigned to a traditional homework condition or a digital support system condition. Both interventions resulted in statistically significant improvements, with large effect sizes, in all the outcome measures at post-treatment, with no significant differences between groups. At 12-month follow-up, these therapeutic gains were maintained, and an improvement was even observed in both conditions, with no significant differences between groups. Additionally, treatment satisfaction predicted efficacy in both groups separately and when the whole group was considered. This is the first study to explore the feasibility an initial efficacy of delivering a therapeutic homework component for AjD through the Internet.

## 1. Introduction

Adjustment disorder (AjD) is one of the most common disorders in clinical practice [1], with prevalence rates of up to 50% in hospital psychiatric consultation settings [2]. In general, the prevalence of the disorder varies depending on the population studied and the diagnostic tools used [3]. Thus, a recent review on AjD reported prevalence rates ranging from 0.9% to 40.0% [4]. It is unclear whether the use of specific diagnostic criteria proposed by ICD-11 affects the estimated incidence of the disorder. However, the scarce literature on this topic suggests that there are no significant differences between using ICD-11 criteria and ICD-10 or DSM-5 criteria [4,5].

AjD is the diagnostic label used to classify patients who develop clinically significant symptoms in response to an identifiable stressor (or multiple stressors) and do not meet diagnostic requirements for any other mental disorder [2,6]. In order to consider that the symptoms appeared as a consequence of a specific stressful event, the onset of both phenomena must be close in time. AjD is usually a transient condition that often resolves on its own within six months if the stressor or its consequences are removed. The greatest contribution to the diagnosis of this disorder was the recent publication of the latest revision of the International Classification of Diseases (ICD-11). The ICD-11 proposes considering preoccupation with the stressor (or its consequences) and failure to adapt to the stressor as the distinguishing features of AjD [6]. Thus, for the first time, AjD acquires the status of a full syndrome with its own set of specific diagnostic criteria. However, at the time the present study began, the classifications mentioned above were not available, and so the DSM-IV-TR [7] and ICD-10 [8], criteria for AjD were followed in this study.

The impact of AjD is another reason this condition deserves clinical attention. Emerging evidence on the course and trajectory of AjD indicates that this problem is a gateway to more severe psychiatric disorders (e.g., [9]), but AjD itself has also been found to be associated with significant negative outcomes [10]. Even though the symptoms of AjD tend to be transient and milder than those presented in other anxiety and affective disorders, they are severe enough to cause great distress, functional impairment, and low quality of life [11,12]. In fact, AjD is one of the main causes of sick leave, and suicide rates are similar to those found in patients with Major Depressive Disorder [13,14].

Despite the relevance of AjD, evidence about effective treatment (both psychological and pharmacological) of the disorder is scarce and inconclusive [3,15,16]. Therefore, there is currently no gold standard treatment approach for AjD. Existing interventions generally focus on reducing the impact of the stressor by providing patients with different coping skills, such as problem solving [17,18] and mindfulness strategies [19].

The cognitive behavioral therapy (CBT) protocol developed by Botella, Baños, and Guillén [20] is one of the few intervention protocols developed specifically for the treatment of AjD that has already provided positive efficacy results [21]. The main goal of this intervention is to promote a positive and adaptive change in the meaning of the stressful event, and it can be applied with virtual reality (VR) support. Specifically, a flexible VR system called *EMMA’s World* was developed to assist in achieving this goal [22]. Results from a randomized controlled trial showed that the protocol has comparable effects with and without the use of the VR system, and the VR group showed slight superiority only at the 12-month follow-up [23]. However, VR can provide additional benefits. For example, indications were found that the use of *EMMA’s World* may have positive effects on the processing of the stressor, making it less aversive [22]. Clearly, this is a good reason to use VR in psychotherapy because, with in vivo exposure, fear is known to be one of the main reasons for attrition in patients suffering from anxiety disorders [24,25]. Furthermore, EMMA’s World has been found to be well accepted by both patients and therapists. It helped to foster motivation in patients, while helping the therapist to apply the treatment [26]. Thus, VR can be also useful as an adjunct tool to enhance the treatment.

The use of between-session homework assignments has also been shown to lead to lower dropout rates [24] and greater treatment response [27,28,29]. Despite existing evidence showing the benefits of using therapeutic homework as part of a psychological intervention, little is known about how to increase homework engagement in psychotherapy.

If therapeutic homework is understood as a way to help patients test and challenge their beliefs and generalize and put into practice the newly acquired skills and knowledge [30], it is reasonable to believe that patients will engage more in homework assignments if they perceive them to be important and useful for their own improvement. Likewise, homework involves autonomous work that has to be done between sessions, and so it resembles a kind of self-help resource. Literature on self-help interventions, such as Internet-delivered interventions, suggests the existence of lower treatment adherence when the intervention represents an important workload for the patient [31]. Internet interventions can be useful to overcome this problem because people can use them in their everyday lives [21]. Thus, administrating therapeutic homework via the internet may help patients to perceive these activities as less boring and demanding. In addition, the Internet makes it possible to create flexible and customizable systems that can fit individual users’ needs. Finally, this way of delivering between-session homework may provide immediate feedback to the patient about the fulfillment of the homework and this can increase the motivation to keep practicing. To our knowledge, no studies evaluating the use of homework delivered via the Internet have been conducted so far.

This work examines the feasibility (doability, initial efficacy and acceptability) of a digital support system to deliver homework via the Internet in the treatment of AjD. Additionally, this study will provide more evidence about the efficacy of the CBT protocol developed by Botella et al. [20] supported by VR, thus contributing to advancing in the treatment of AjD. Evidence-based interventions are needed in order to prevent the chronicity of the disorder and the worsening of clinical symptoms [10,32]. Therefore, a pilot randomized clinical trial (RCT) was conducted comparing two groups that received the same CBT protocol supported by VR. The only difference was the way they practiced the between-session homework (in the traditional format or using the Internet). Because the previous randomized controlled trial showed the superiority of the CBT intervention over the waiting list control condition [33], no other control group was included. Finally, the role of treatment satisfaction in clinically significant change at post-treatment and 12-month follow-up is also explored.

## 2. Method

### 2.1. Design

**Figure 1:**
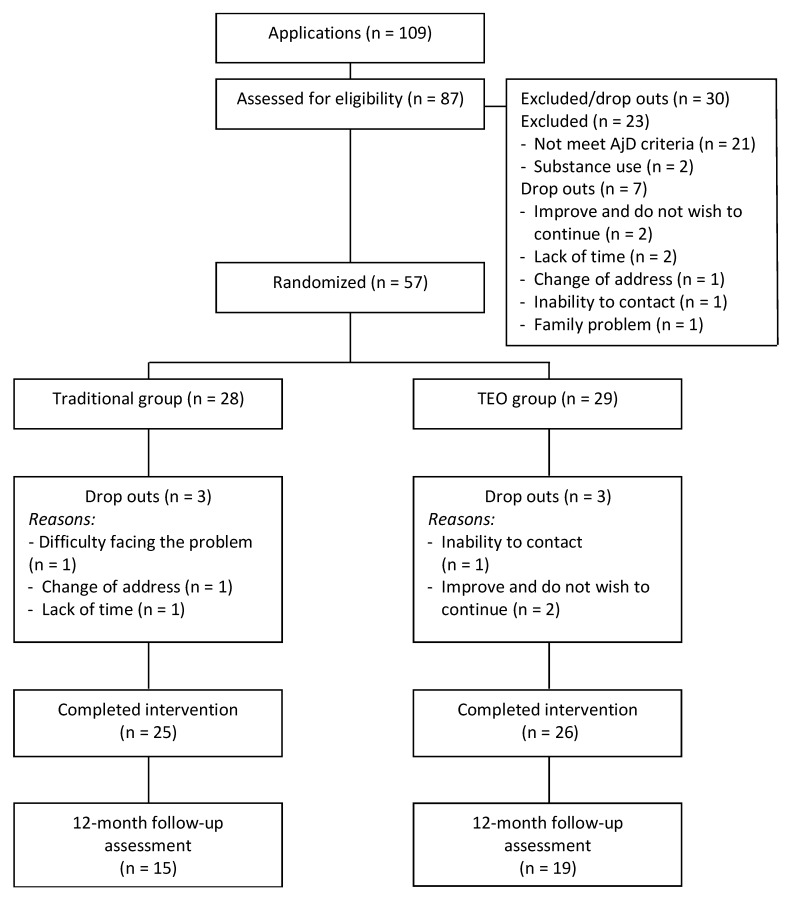
Flowchart of participants and dropout rates.

This study is a two-arm exploratory pilot RCT with three assessment moments (pre-treatment, post-treatment, and 12-month follow-up). Participants were randomized into two experimental groups: (1) the traditional homework condition; and (2) the *Terapia Emocional Online* (TEO) system homework condition. TEO is a digital support system that allows therapists to create and send personalized homework sessions to patients via the Internet so that they can receive assignments and practice online. The difference between the two experimental conditions was the way participants carried out the homework assignments: in a traditional way or using an online system. The power analysis conducted with G*Power indicated that a total sample of *N* = 30 (15 participants per group) was enough to detect a between-group effect size of 1.26 reported in [23], with 90% power and assuming α = 0.05. This pilot RCT was conducted following the Consolidated Standards of Reporting Trials (CONSORT, http://www.consort-statement.org) statement, and registered on clinicaltrials.gov (NCT02452411), and it received approval from the Ethics Committee of Universitat Jaume I. In the present study, results related to treatment efficacy are presented. Session-to-session efficacy data from the homework assignment component and the patients’ preferences and opinions about this component in both formats (Traditional vs TEO) are in preparation for publication elsewhere. Figure 1 shows the flowchart of the study.

### 2.2. Participants

This study was conducted at the Emotional Disorders Clinic at Universitat Jaume I in Castellón (Spain). Participant recruitment was carried out through advertisements (e-mail, posters, radio, and press) about the study held in our clinic. Potential participants were also derived from the Emotional Disorder Clinic. Inclusion criteria were: age between 18 and 65 years old; meeting AjD criteria according to the DSM-IV-TR [7] and ICD-10 [8]; and access to a computer and Internet. Exclusion criteria were: current alcohol or drug dependence or abuse; psychosis or a severe personality disorder; a severe organic illness that makes it impossible to apply the treatment program; risk of suicide; and currently receiving another psychological treatment for the same problem. Receiving pharmacological treatment was not an exclusion criterion, but any increase in the medication during the study period led to the participant’s exclusion from subsequent analyses.

A total of 57 participants were eligible to participate, completed baseline assessment, and were therefore included in the analysis. Of these participants, 28 were allocated to the Traditional group and 29 to the TEO group. Participants’ ages ranged from 18 to 54 years (mean 30.07, SD 10.47). The majority of the sample were women (75.4%) and had a university education (73.7%). Only 18.2% of the participants were taking medication. This medical prescription was maintained or reduced during the study. See Table 1 for a more detailed description of the sample.

### 2.3. Measures

#### 2.3.1. Diagnostic Instrument

*Diagnostic Interview for Adjustment Disorders* [34]. This interview was developed taking into consideration the diagnostic criteria for AjD included in the DSM-IV-TR [7], the ICD-10 [8], and the *Structured Clinical Interview for DSM-IV (SCID-CV)* [35]. No adjustments were made in the interview after the publication of the DSM-5 [2] because the only change included in the latest version of this manual involved the classification of AjD in the category of *Trauma and Stress Related Disorders*. It is a semi-structured interview for the diagnosis of AjD and includes two main parts: (1) evaluation of past or current stressful events from a list of 62; (2) evaluation of the presence and severity of 28 symptoms related to AjD on a scale from 0 (*Not at all*) to 8 (*Extremely severe*). Moreover, several items were included to detect the need to perform differential diagnoses with Post-traumatic Stress Disorder and Generalized Anxiety Disorder, and to determine that the symptoms were not induced or aggravated by a medical condition or substance use.

#### 2.3.2. Primary Outcomes

*Beck Depression Inventory - Second Edition* (BDI-II) [36], validated in the Spanish population [37]. The BDI-II measures characteristic attitudes and symptoms of depression. It contains 21 items rated on a scale from 0 to 3, and it has well-established psychometric properties (Cronbach’s alphas from 0.76 to 0.95 and test-retest reliability of around 0.8).

*Inventory of Stress and Loss (ISL)* [33]. This questionnaire is an adaptation of the Complicated Grief Inventory [38] and it explores the degree to which the person / situation interferes with the individual’s life. It is a 17-item instrument with a rating scale from 0 to 4 (0 = never; 4 = always). The Spanish version of the ISL has demonstrated high internal consistency (0.92 and 0.85 for the non-clinical group and the AjD group, respectively).

#### 2.3.3. Secondary Outcomes

*Positive and Negative Affect Scale (PANAS)* [39], validated in the Spanish population [40] PANAS is a 20-item measure of two primary dimensions of mood: Positive Affect (PA; 10 items) and Negative Affect (NA; 10 items). Items are rated on a scale from 1 to 5 (1 = very slightly or not at all; 5 = extremely), and the total range for each scale (PANAS-PA and PANAS-NA) is from 10 to 50. The Spanish version of the PANAS has demonstrated high internal consistency in college students (α = 0.89 for PA and 0.91 for NA in women, and 0.87 for PA and 0.89 for NA in men).

#### 2.3.4. Treatment Satisfaction

*Treatment Satisfaction Scale*. This instrument was adapted by our research group from Borkovec and Nau [41]. It is a self-report inventory that measures satisfaction with treatment. The scale has a total of 5 items assessing: the perceived logic of the treatment; satisfaction with the treatment; the recommendation of the treatment to a friend with a similar problem; the perceived usefulness of the treatment for other psychological problems; and its usefulness for the particular problem. The 5 items are rated from 1 to 10 (1 = not at all; 10 = highly). This adaptation has been used in previous studies [42,43,44].

### 2.4. Treatment

#### CBT AjD Protocol

A slightly modified version of the AjD protocol by Botella et al. [20] was administered to the participants in this study. The aim of the treatment was to help the AjD patients adapt to the stressful event and its negative consequences, and improve their capacity to face problems and learn from them after the event. The number of sessions for the treatment varied from 6 to 8, with each session lasting approximately 90 min. The patients received one session per week and had to do homework assignments between the sessions.

The therapeutic components of the treatment were the following:

*Psychoeducation*: This component provides information about the common reactions to stressful events, the adaptive and maladaptive ways to face a problem, and what maintains the symptoms of AjD.

*Exposure, elaboration/processing of the stressful event*: The aim of this therapeutic component is to give the person tools to elaborate, find alternative meanings, and learn from the stressful experience they had. For this purpose, based on Neimeyer’s [45] suggestions for the treatment of grief, the *Book of Life* is used. The patients elaborate chapters for this book with meaningful situations related to the adverse event. They add symbols and pictures related to the event in their book. While doing this work, patients are given the instruction to be aware of emotions, thoughts, and behaviors, without judging them, during the elaboration process. The application of this component was supported by an adaptive VR system, EMMA’s World, which includes five predefined scenarios (a desert, an island, a forest, a snow-covered town, and meadows) and different symbols (such as images, 3D objects, sounds, and music). Furthermore, the therapist can modify several aspects of these scenarios in real time (e.g., the weather, the time of day, the light, etc.). The objective is to adapt the VR system’s contents to the patient’s needs, with the aim of reflecting and activating emotions and thoughts related to the particular stressful event. Patients can choose the elements of the VR system that best reflect the negative event they experienced. At the start and end of each session, the therapist asks the patient if he/she wants to change anything in the virtual world, and in the next session the environment is the same as in the previous one. For a more detailed description of EMMA’s World and technical aspects, see [21,22,45,46,47].

*In vivo exposure*: This technique is used to confront the situations or people related to the stressful event that patients are avoiding.

*Positive psychology strategies*: The strategies included were problem acceptance training [48]; my best virtues or strengths [49]; and a heuristics exercise where the patients have to choose a proverb, statement, or life guideline that they find helpful in maintaining and promoting the changes achieved in their lives.

*Relapse prevention*: The aim of this component is to maintain and promote the changes achieved in therapy in the future, reviewing all the objectives achieved so far and summarizing the things they learned during the therapy. A more detailed description of the treatment protocol can be found in Quero et al. [23].

**Figure 2:**
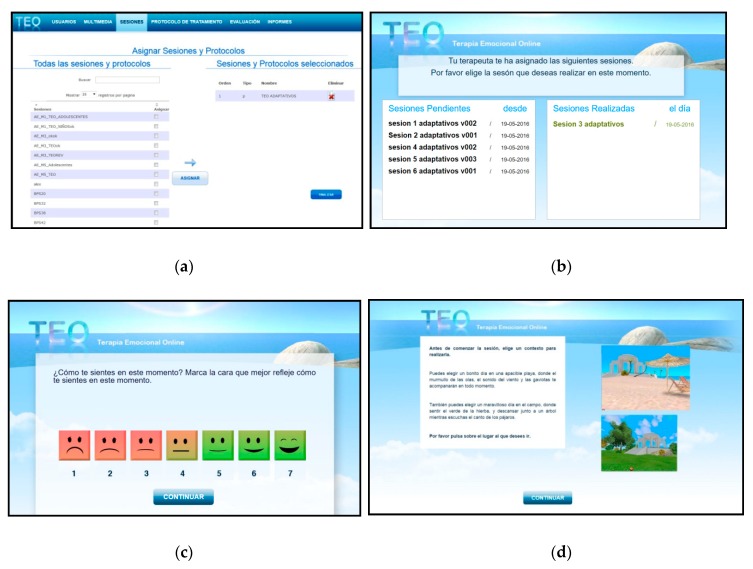

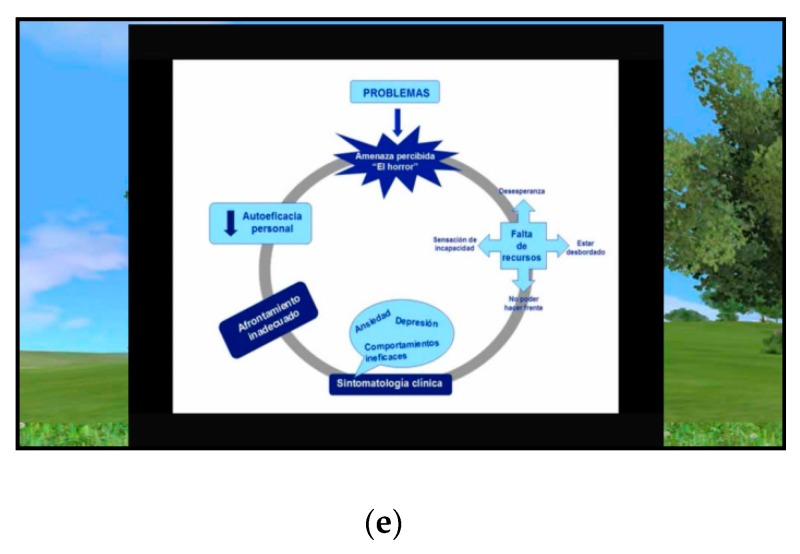
TEO system: therapist platform—(**a**) session assignment; patient platform—(**b**) view of the assigned sessions, (**c**) assessment online, (**d**) choice of the virtual environment, (**e**) view of therapeutic material.

Finally, the AjD manualized protocol includes a *homework assignment component*. Table 2 shows a brief description of the homework session protocol for AjD. After each treatment session, participants completed homework sessions to reinforce the therapeutic components learned in the therapy sessions. This therapeutic component was applied in two ways:

(1) In the Traditional condition, the homework component is composed of: (1) reading material in the form of brief manuals as reminders of the different therapeutic contents; (2) writing materials in the form of self-registers and exercises in order to practice the contents seen in the therapy sessions in real life; and (3) therapy session audio recordings in order to practice the elaboration/processing of the stressful event.
(2) In the TEO condition, a digital support system was used. TEO is a completely open web-based technology that allows therapists to create and easily send personalized therapeutic material to patients via the Internet (http://www.teo.uji.es). The system has a database with media contents (audio, images, videos, and texts), and the therapists can access these materials and combine them to create homework sessions. TEO is a therapeutic tool developed to complement therapy. In the present study, it is used to administer the homework sessions in the period between the treatment sessions. Therefore, the system is designed to consolidate, support, and improve the therapeutic intervention by assigning homework that can be practiced at home via the Internet. Moreover, through this program, several clinical variables (e.g., mood state or positive and negative emotions) can be assessed before and after each homework session. The therapist can access the reports resulting from a patient’s activity: assessment, content, assigned sessions, completed sessions, dates, etc. The TEO system includes two platforms with different functions:
  *Therapist platform*: In this platform, the therapist can manage and administer the users and the results of each user’s treatment and create sessions and customize homework protocols using multimedia materials included in the TEO multimedia base (pictures, texts, narratives, music, and videos). These multimedia elements cover the clinical needs of each patient and work on all the therapeutic components included in the AjD protocol. The therapist can view the content created and assign homework sessions (see Figure 2a). Moreover, assessment protocols are assigned to the users and composed of brief questionnaires assessing key clinical variables. The therapist can download the results of these evaluations in order to monitor each patient.
  *Patient platform*: The main function of the patient platform is to allow the user to receive and view the session assigned and sent by the therapist (see Figure 2b). First, the patients can complete the pre-session assessment protocol (see Figure 2c) and choose a virtual environment (a beach or a forest environment) to go to throughout the homework session (see Figure 2d). This virtual environment also appears at the end of each session, so that the patients can move around to reflect on the session and their experience. Then, they can complete the post-assessment protocol and repeat the session as many times as necessary. In the present study, we used the TEO system to reinforce the treatment components described above. To do so, we designed a homework session component using audios, videos, and images that were available in the TEO system database. Specifically, the TEO system allows access to all the therapeutic material available in EMMA’s world (symbols, audios, and images), with the aim of continuing with the processing/elaboration treatment component from home. Figure 2e presents an example of therapeutic material included in a specific TEO homework session for the treatment of AjD.

### 2.5. Procedure

Participants who were eligible for the study were informed about the project and signed the informed consent. After that, they underwent two assessment sessions lasting 1.5 h each in order to confirm the AjD diagnosis and establish the therapeutic goals. Then, participants were randomly allocated to each condition (Traditional or TEO homework condition) by an independent researcher, based on a computer-generated list created by the Random Allocation Software, version 1.0. After the allocation, participants received the 6-8 CBT AjD protocol, with the only difference being the way the homework assignment component was administered. Participants in the traditional condition used the CBT AjD manualized protocol described above (including reading, audio, and writing materials to complete the tasks), whereas participants in the TEO condition practiced the component through the online TEO system. Once the treatment was over, all the participants were assessed again at post-treatment and at 12-month follow-up. Seven therapists participated in the study. They had a PhD or Master’s degree in Clinical Psychology. All of them were trained in VR techniques and CBT programs for Emotional Disorders. In addition, they had received training in this treatment protocol from senior clinicians. They were supervised by senior clinicians with PhDs in weekly sessions.

### 2.6. Data Analyses

Baseline between-group differences were explored using independent-sample *t* tests or chi-square analyses, depending on whether continuous or categorical data were analyzed.

Treatment effectiveness was estimated using linear mixed models, an intent(ion)-to-treat analysis based on all available observations [50]. The analyses were conducted separately for each outcome measure, introducing time, treatment group, and the time by group interactions as fixed factors within the model. Within- and between-group changes from baseline to post-intervention and from post-intervention to 12-month follow-up were compared using planned contrasts. The magnitude of the intervention’s effect was estimated using Cohen’s *d* effect sizes. Following the existing literature, effect sizes of 0.2, 0.5, and 0.8 were considered small, moderate, and large, respectively [51]. Within-group effect sizes were calculated between baseline and post-treatment mean scores, and between post-treatment and 12-month follow-up mean scores.

The clinical significance of the observed changes in the outcome measures was determined by calculating reliable change indexes [52] between baseline and post-treatment and between baseline and follow-up scores. This made it possible to determine the proportion of participants who showed reliable improvement after the intervention.

Finally, linear regression analyses were conducted to explore the role of treatment satisfaction in the clinically significant change experienced from baseline to post-treatment and from baseline to follow-up.

All statistical analyses were conducted using the statistical package IBM SPSS Statistics, version 25.0 for Windows (IBM Company, Madrid, Spain), and following the current SPIRIT [53,54] and CONSORT [55] guideline recommendations.

## 3. Results

### 3.1. Participant Flow and Attrition

The recruitment started on January 2013 and ended on December 2015. Initially, 109 people were interested in the study (see flowchart in Figure 1), and 87 of them were assessed for eligibility criteria. In this phase, 30 participants were excluded from the study. Finally, 57 participants were included in the study and randomly allocated to each experimental condition (Traditional condition, n = 28; TEO condition, n = 29). In the Traditional group, 25 of the 28 participants finished the intervention and completed the post-treatment assessment (89.3%). In the TEO group, 26 of the 29 participants completed the post-treatment assessment (89.7%). Of the six participants who did not finish the CBT AjD protocol, five reported the following reasons for their dropout: remission of symptoms (N = 2), lack of time (N = 1), change of place of residence (N = 1), and difficulties facing the stressor (N = 1). One participant did not give any reason for dropping out. At 12-month follow-up, 15 participants from the Traditional group (53.6%) and 19 participants from the TEO group (65.5%) could be reached for the assessment. Little’s MCAR test was not significant for the post-treatment (χ^2^ = 28.10, ρ = 0.212) or follow-up assessment (χ^2^ = 10.66, *p* = 0.222).

### 3.2. Baseline Data and Participants’ Characteristics

No statistically significant differences were found between the experimental groups before treatment on any of the sociodemographic variables, except for marital status (χ^2^ (2) = 6.23, ρ = 0.044) (see Table 1). Furthermore, no significant differences were found on the primary and secondary outcomes at baseline.

### 3.3. Treatment Efficacy

Table 3 and Table 4 present the results on treatment effectiveness at post-treatment and 12-month follow-up, respectively. At post-treatment, within-group comparisons revealed significant improvements in participants in both study groups on all outcomes, with large within-group effect sizes. Small and non-significant between-group differences were found. In both groups, similar proportions of participants showed clinically significant changes in self-report scores, with no significant between-group differences: BDI (Traditional: 91.7% vs. TEO: 84.6%; χ^2^ = 5.00, ρ = 0.082), SLI (Traditional: 78.3% vs. TEO: 76.9%; χ^2^ = 0.94, ρ = 0.626), positive affect (Traditional: 44.0% vs. TEO: 48.0%; χ^2^ = 0.09, ρ = 0.956), negative affect (Traditional: 48.0% vs. TEO: 48.0%; χ^2^ = 1.04, ρ = 0.595).

Twelve months after the end of the intervention, the clinical changes achieved at post-treatment were maintained and even improved (see Table 4). Both groups presented significant reductions in symptoms related to adjustment disorder and a significant increase in positive affect. The Traditional group also reported a significant reduction in negative affect, whereas participants from the TEO group experienced a significant reduction in depressive symptoms. Within-group effect sizes were larger in the Traditional group. Between-group differences were small and not statistically significant. Percentages of patients who showed reliable improvements from baseline did not differ between the two treatment groups: BDI (Traditional: 93.3% vs. TEO: 84.2%; χ^2^ = 1.59, ρ = 0.453), SLI (Traditional: 93.3% vs. TEO: 100.0%; χ^2^ = 1.31, ρ = 0.253), positive affect (Traditional: 60.0% vs. TEO: 63.2%; χ^2^ = 0.82, ρ = 0.664), negative affect (Traditional: 60.0% vs. TEO: 52.6%; χ^2^ = 0.19, ρ = 0.667).

### 3.4. Treatment Satisfaction

In the Traditional group, participants’ mean score on the Treatment Satisfaction Scale was 49.08/50.00 compared to 49.22/50.00 in the TEO group at post-treatment and 45.32/50.00 compared to 45.13/50.00 at 12-month follow-up. No significant between-group differences were found (post-treatment: t = −0.06, ρ = 0.956; follow-up: t = 0.18, ρ = 0.858). Descriptive statistics for each item are presented in Table 5.

Regression analysis revealed that each participant’s total score on the Treatment Satisfaction Scale predicted his or her response to the received treatment (see Table 6). At post-treatment, greater treatment satisfaction predicted 19% of the reduction in BDI scores in the Traditional group. In the TEO group, treatment satisfaction contributed to the clinically significant change on the SLI and the negative affect subscale of the PANAS (22% and 21%, respectively).

When no distinction was made between the two experimental groups, treatment satisfaction explained 10% of the variance in SLI scores, and 12% of the variance in BDI and the positive affect subscale of the PANAS. At 12-month follow-up, 39% of the variance in SLI and 47% of the variance in the positive affect subscale of the PANAS in the Traditional group was explained by treatment satisfaction. Combined analysis revealed the influence of treatment satisfaction on the change in the SLI and positive affect scale scores (20% and 14%, respectively).

## 4. Discussion

The main objective of the present work was to examine the feasibility of a digital support system to deliver homework via the Internet in the treatment of AjD. Results obtained in the pilot RCT showed that both interventions (the one that applied the homework component in the traditional way and the one that used the TEO system) resulted in statistically significant improvements with large effect sizes in all the outcome measures at post-treatment, with no differences found between groups. Additionally, high percentages of participants reached clinically significant change, mainly on depression and stress and loss of symptoms, again with no differences between groups. These data are consistent with those obtained in previous studies [21,23] where the same treatment protocol was applied using the traditional homework format. Therefore, the present study provides additional evidence about the efficacy of the AjD protocol by Botella et al. [20]. These results are also consistent with the conclusions reported in systematic reviews specifically evaluating the usefulness of virtual reality for stress-related disorders (e.g., [56,57,58]).

In the long-term, these therapeutic gains were maintained, and an improvement was even observed in stress and loss and positive affect in both conditions. When considering each condition separately, an additional improvement was produced in negative affect in the traditional group, whereas in the TEO group this additional improvement was found in depressive symptoms. Again, no differences between groups were found. Furthermore, at 12-month follow-up, higher percentages of participants reached clinically significant changes in all outcome measures, with no differences between groups. This result showing the maintenance of the therapeutic achievements after 1 year was also obtained in the previous study conducted with AjD and complicated grief patients [23].

A second aim of this study was to explore the role of treatment satisfaction in clinically significant change in the short- and long-term. Participants in both conditions reported very high levels of satisfaction at post-treatment and follow-up, with no significant differences between them. Therefore, the results show that delivering the homework component through the internet was well accepted by participants allocated to this condition and they practiced the homework as the participants did in the traditional condition. Additionally, regression analyses showed that treatment satisfaction predicted treatment efficacy in both groups separately, Traditional and TEO, and when the whole group was considered. These data are consistent with those obtained in the virtual and augmented reality field, showing that a treatment not only has to be effective, but also well-accepted by patients (e.g., [22,43,59]. No study exploring the specific relationship between treatment satisfaction and treatment outcomes for AjD was found in the literature.

The main contribution of the present study is to bring to light that it is possible to deliver digitally a very important component, within the psychological treatment field in general and the CBT approach in particular, that is, therapeutic homework (e.g., [60]). To our knowledge, this is the first study to explore the feasibility an initial efficacy of delivering a therapeutic homework component for AjD through the Internet. As mentioned in the introduction, the use of a digital support system like TEO can help patients to perceive these activities as less boring and demanding and, consequently, increase adherence to them. This issue is important, considering the previous evidence about the use of between-session homework assignments to decrease dropout rates [24] and achieve greater treatment response [27,28,29]. Attrition rates in the present study showed no significant differences between conditions at post-treatment or follow-up, although more participants in the TEO condition completed the follow-up assessment. However, the specific attrition rate for the homework assignment component and the frequency with which homework was completed were not assessed in the present study. Therefore, future studies are needed to test whether the use of a system like TEO could indeed reduce attrition rates and increase adherence rates to homework. Moreover, data on preferences and opinions about this component in terms of the efficiency of TEO system are of interest.

Another benefit of using the Internet to deliver interventions is greater dissemination, reaching different target groups [61]. Particularly, a flexible tool like the TEO system can be used in a blended mode, complementing therapy, or in a tele-assistance mode as self-administered therapy. It increases the intervention possibilities, making it more accessible for all the potential users. Enhancing the accessibility of psychological treatments is one of the main challenges in general mental health [62,63,64]. In addition, the TEO system offers great adaptability and flexibility in adjusting the therapeutic homework to each patient’s characteristics and needs. In other computerized treatment programs, the therapeutic sessions are closed and the same for all users. However, this tool allows clinicians to configure personalized therapeutic sessions (which are modifiable) that can potentially be useful for any emotional disorder. Finally, this web-based system allows patients to practice at home what they have worked on during the therapy sessions in a self-applied way. In this way, they not only receive treatment in the consultation room, but also in the period between sessions, providing the patient with a more active role and more involvement in the whole treatment process, as well as immediate and continuous feedback about the fulfillment of the homework, which can increase the patient’s motivation to keep practicing.

Some limitations of this study should be highlighted. First, the diagnostic interview for AjD used in the present study has not been validated yet. Second, although the sample size in this study was larger than the one used in a previous study [23] in which pre-post differences with the same CBT protocol for AjD were found with large effect sizes, the present study is exploratory and we cannot assure that we have enough statistical power. Third, participants were mainly women and had university degrees, compromising the generalization of the results.

A future recommendation in the field to improve psychological interventions for AjD is to use the Internet to apply the whole intervention, and not only the homework assignment component. Our team has already developed an Internet-based program called *Trastornos Adaptativos Online* (TAO) which has provided preliminary evidence of its acceptability and usability [65]. In addition, preliminary efficacy data on a modular self-applied Internet-based intervention (BADI) for the treatment of AjD symptoms have been reported [66]. These advances will make it possible to avoid problems of access to psychological treatments by people who need help and cannot receive it [63,67]. This is a key advantage, considering the high prevalence of AjD [3,68], the great distress and functional impairment this disorder can cause in people who suffer from it [11,12] and its potential life-threatening features [14].

## 5. Conclusions

First, this study confirms that the homework component can be successfully applied in a conventional way and through digital methods. Second, this study also confirms that the majority of patients improve during treatment for AjD. Large effect sizes were found at post-treatment for primary outcomes (depression and stress and loss symptoms) and secondary measures (positive and negative affect), with no significant differences between groups. Moreover, this improvement reached clinical significance in a high percentage of patients on the primary outcome measures, again with no significant differences between groups. Additionally, these therapeutic gains were maintained in the long term, and further improvement was even seen in both conditions. Finally, this study adds evidence about the role of treatment satisfaction in treatment outcomes in AjD. Participants in both conditions were highly satisfied with the intervention, and treatment satisfaction predicted efficacy in both groups.

## Figures and Tables

**Table 1:** Participant characteristics.

	Traditional Group (N = 28)	TEO Group (N = 29)
Age, mean (SD)	31.71 (11.27)	28.48 (9.55)
Gender, n (%)		
Male	6 (21.4%)	8 (27.6%)
Female	22 (78.6%)	21 (72.4%)
Education, n (%)		
Elementary or secondary	10 (35.7%)	5 (17.2%)
University	18 (64.3%)	24 (82.8%)
Marital status, n (%)		
Single	16 (57.1%)	25 (86.2%)
Married/partnered	8 (28.6%)	2 (6.9%)
Widowed/divorced	4 (14.3%)	2 (6.9%)
Medication, n (%)		
Yes	7 (25.9%)	3 (10.7%)
No	20 (74.1%)	25 (89.3%)
Months since symptom onset, mean (SD)	29.00 (44.23)	20.03 (31.41)

**Table 2:** Homework contents

	Homework
1	Treatment explanation
	The impact of stressful events
	Cognitive Model of AjD (part I)
	Logic of using the Book of Life and EMMA’s world
2	Explanation if the exposure and elaboration techniques
	Breathing exercises (optional)
	Elaboration of stressful event practice
	Self-exposure tasks to the first item in the exposure hierarchy
3	Elaboration practice
	“Positive” meaning of problems (Part I)
	Breathing exercises (optional)
	Self-exposure tasks
4	Elaboration practices
	“Positive” meaning of problems (Part II)
	Self-exposure tasks
5	Elaboration practices
	Cognitive Model of AjD (Part II)
	“Letter to my future self”
6	Practice of a new “metaphorical description” of the stressful event in EMMA’s world
	Choose of search for a heuristic/proverb to apply in everyday situations

**Table 3:** Descriptive statistics and estimated within- and between-group differences at post-treatment.

	BL Mean (SD)	Post Mean (SD)	Baseline vs. Post-Treatment			
			Within-Group d [95% CI]	Within-Group Comparison	Between-Group d [95% CI]	Between-Group Comparison
BDI						
Traditional group	24.18 (8.91) (n = 28)	4.91 (3.93) (n = 24)	2.09 [1.34; 2.85]	F_1, 50.61_ = 119.80, ρ = 0.000 **	0.36 [−0.92; 0.20]	F_1, 91.39_ = 0.52, ρ = 0.473
TEO group	22.48 (10.86) (n = 29)	7.19 (7.68) (n = 26)	1.37 [0.81; 1.92]	F_1, 49.75_ = 86.82, ρ = 0.000 **		
SLI						
Traditional group	35.38 (11.97) (n = 28)	15.87 (11.18) (n = 24)	1.58 [0.95; 2.20]	F_1, 49.75_ = 75.07, ρ = 0.000 **	0.11 [−0.67; 0.44]	F_1, 85.79_ = 0.03, ρ = 0.862
TEO group	37.22 (10.58) (n = 29)	17.15 (11.43) (n = 26)	1.84 [1.18; 2.50]	F_1, 49.02_ = 102.87, ρ = 0.000 **		
PANAS +						
Traditional group	22.64 (6.95) (n = 28)	32.68 (7.34) (n = 25)	1.40 [−1.97; −0.82]	F_1, 49.84_ = 41.04, ρ = 0.000 **	0.30 [-0.25; 0.86]	F_1, 90.08_ = 0.84, ρ = 0.362
TEO group	21.91 (8.45) (n = 29)	30.20 (8.63) (n = 25)	0.95 [−1.44; −0.46]	F_1, 50.49_ = 31.50, ρ = 0.000 **		
PANAS -						
Traditional group	27.30 (7.69) (n = 28)	16.84 (5.96) (n = 25)	1.32 [0.76; 1.88]	F_1, 47.44_ = 43.76, ρ = 0.000 **	0.11 [−0.66; 0.45]	F_1, 93.73_ = 0.01, ρ = 0.909
TEO group	25.86 (8.33) (n = 29)	17.48 (5.75) (n = 25)	0.97 [0.48; 1.47]	F_1, 48.18_ = 30.93, ρ = 0.000 **		

** *p* < 0.01. BL = Baseline; Post = Post-treatment; SD = Standard Deviation; d = Cohen’s d effect size; CI = Confidence Interval; BDI = Beck Depression Inventory; SLI = Stress and Loss Inventory; PANAS+ = Positive and Negative Affect Scale, positive affect subscale; PANAS- = Positive and Negative Affect Scale, negative affect subscale.

**Table 4:** Descriptive statistics and estimated within- and between-group differences at 12-month follow-up.

9	Post Mean (SD)	Fup Mean (SD)	Post-Treatment vs. Follow-Up			
			Within-Group d [95% CI]	Within-Group Comparison	Between-Group d [95% CI]	Between-Group Comparison
BDI						
Traditional group	4.91 (3.93) (n = 24)	2.00 (2.17) (n = 15)	0.70 [0.11; 1.29]	F_1, 34.22_ = 3.91, ρ = 0.056	0.44 [−1.13; 0.24]	F_1, 78.26_ = 1.38, ρ = 0.244
TEO group	7.19 (7.68) (n = 26)	4.05 (5.69) (n = 19)	0.39 [−0.09; 0.87]	F_1, 32.25_ = 5.07, ρ = 0.031 *		
SLI						
Traditional group	15.87 (11.18) (n = 24)	5.33 (4.17) (n = 15)	0.89 [0.26; 1.52]	F_1, 31.14_ = 19.28, ρ = 0.000 **	0.43 [−1.12; 0.25]	F_1, 76.17_ = 1.18, ρ = 0.282
TEO group	17.15 (11.43) (n = 26)	8.09 (7.49) (n = 19)	0.76 [0.23; 1.29]	F_1, 29.59_ = 14.37, ρ = 0.001 **		
PANAS +						
Traditional group	32.68 (7.34) (n = 25)	39.13 (8.40) (n = 15)	0.83 [−1.45; −0.21]	F_1, 41.46_ = 7.67, ρ = 0.008 **	0.40 [−0.28; 1.09]	F_1, 79.68_ = 1.96, ρ = 0.165
TEO group	30.20 (8.63) (n = 25)	35.47 (9.25) (n = 19)	0.58 [−1.09; −0.08]	F_1, 37.36_ = 5.40, ρ = 0.026 *		
PANAS -						
Traditional group	16.84 (5.96) (n = 25)	13.73 (2.89) (n = 15)	0.49 [−0.06; 1.05]	F_1, 36.48_ = 4.23, ρ = 0.047 *	0.45 [−1.14; 0.23]	F_1, 79.34_ = 1.21, ρ = 0.276
TEO group	17.48 (5.75) (n = 25)	15.63 (4.83) (n = 19)	0.31 [−0.16; 0.78]	F_1, 32.78_ = 1.56, ρ = 0.220		

* *p* < 0.05; ** *p* < 0.01. Post = Post-treatment; Fup = 12-month Follow-up; SD = Standard Deviation; d = Cohen’s d effect size; CI = Confidence Interval; BDI = Beck Depression Inventory; SLI = Stress and Loss Inventory; PANAS+ = Positive and Negative Affect Scale, positive affect subscale; PANAS- = Positive and Negative Affect Scale, negative affect subscale.

**Table 5:** Descriptive statistics for Treatment Satisfaction Scale.

	Post *M (SD)*		Fup *M (SD)*	
	Traditional Group	TEO Group	Traditional Group	TEO Group
Treatment was logical	8.80 (0.82)	8.96 (1.00)	9.00 (1.16)	9.00 (0.91)
Treatment was satisfactory	9.24 (0.78)	9.13 (1.15)	9.46 (0.97)	9.11 (1.18)
Would recommend the treatment	9.44 (0.71)	9.33 (0.87)	9.31 (0.86)	9.39 (0.85)
Treatment was useful for the problem	9.28 (0.68)	9.00 (1.14)	9.54 (0.97)	9.00 (1.33)
Treatment was useful for other problems	8.56 (1.12)	8.71 (1.12)	8.77 (1.01)	8.89 (1.37)

**Table 6:** Estimation of the variance of clinically significant change attributable to patients’ satisfaction with the received treatment.

	Treatment Satisfaction at Post-Treatment				Treatment Satisfaction at 12-Month Follow-Up			
	R^2^	β	t	ρ	R^2^	β	t	ρ
BDI								
Traditional group	0.19	−0.47	−2.50	0.021 *	0.20	−0.51	−1.98	0.074
TEO group	0.08	−0.35	−1.73	0.099	0.00	−0.25	−1.03	0.319
Total	0.12	−0.37	−2.72	0.009 **	0.08	−0.34	−1.92	0.065
SLI								
Traditional group	−0.05	−0.03	−0.14	0.892	0.39	−0.67	−2.97	0.013 *
TEO group	0.22	−0.50	−2.72	0.013 *	0.08	−0.37	−1.57	0.136
Total	0.10	−0.34	−2.45	0.018 *	0.20	−0.48	−2.92	0.007 **
PANAS+								
Traditional group	0.11	0.38	1.99	0.059	0.47	0.72	3.42	0.006 **
TEO group	0.11	0.39	1.97	0.062	−0.01	0.22	0.90	0.380
Total	0.12	0.37	2.73	0.009 **	0.14	0.41	2.42	0.022 *
PANAS-								
Traditional group	−0.04	−0.02	−0.08	0.938	0.05	−0.36	−1.29	0.225
TEO group	0.21	−0.49	−2.64	0.015 *	0.03	−0.30	−1.25	0.230
Total	0.05	−0.26	−1.82	0.075	0.07	−0.32	−1.81	0.080

* *p* < 0.05; ** *p* < 0.01.
